# Optical Genome Mapping in MDS and AML as tool for structural variant profiling—comment and data update on Yang et al.: “High-resolution structural variant profiling of myelodysplastic syndromes by optical genome mapping uncovers cryptic aberrations of prognostic and therapeutic significance”

**DOI:** 10.1038/s41375-022-01763-2

**Published:** 2022-11-24

**Authors:** Deepak B. Vangala, Verena Nilius-Eliliwi, Wanda M. Gerding, Roland Schroers, Huu Phuc Nguyen

**Affiliations:** 1 Dept. of Medicine, Center for Hemato-Oncological Diseases, University Hospital Knappschaftskrankenhaus, Ruhr-University, Bochum, Germany; 2 Human Genetics, Ruhr-University Bochum, Bochum, Germany

**Keywords:** Cytogenetics, Genetics research

## To the Editor:

Yang et al. recently published their work entitled “High-resolution structural variant profiling of myelodysplastic syndromes by optical genome mapping uncovers cryptic aberrations of prognostic and therapeutic significance” in leukemia [1]. The authors describe a high concordance in 96/99 cases of detected structural variants by routine diagnostics, most importantly chromosomal banding analysis (CBA), and optical genome mapping (OGM) as a whole genome method for structural variant profiling (SVP). More importantly, in 34% of the 101 patients included in the analysis, cryptic variants could be clarified and the risk score according to R-IPSS was changed by additional information obtained by OGM in 17% of patients.

These results and the approach chosen are well in line with the data published earlier this year by our group [2]. Although focusing on acute myeloid leukemia (AML), we also included a subgroup of six myelodysplastic syndrome (MDS) patients, three of them with excessive blasts I and three with excessive blasts II, or MDS/AML according to the recently updated international consensus classification [3]. Furthermore, 8 of 21 AML patients had myelodysplasia-related changes.

Currently, and updating our previous data, our cohort consists of 42 patients (35 AML and 7 MDS, Table 1). Of 42 patients, a conventional diagnostic approach by cytogenetics (CBA; fluorescence in situ hybridization (FISH) and Copy-number variation (CNV)-microarrays where indicated) was able to detect variants in 26 cases. All but three of these variants could also be seen by OGM, the latter below the applied threshold of 5% variant allele frequency (VAF) and in high-repetitive regions such as around telomers and centromeres [4]. In 27 cases (64%), we could clarify or add additional information by using OGM as a non-DNA dependent amplification based whole genome method. For MDS patients this number was similar with 4/7 cases, where the karyotype could be redefined by utilizing OGM (Table 2).

**Table 1:** Clinical characteristics of the study population.

Gender	
Female	18
Male	24
Age median/years (range)	61 (21–75)
Diagnosis	
AML (*n*)	23
AML from prior MDS /AML-MRC (n)	12
MDS (*n*)	7
Risk classification	
ELN 2017	
Good (*n*)	6
Intermediate (*n*)	13
Poor (*n*)	15
IPSS-Score	
Very high (*n*)	3
High (*n*)	3
Intermediate (*n*)	1

Due to the inclusion prior to the novel world health organization (WHO) classification and European Leukemia Net (ELN) risk groups, here WHO2016 and ELN 2017 classifications are still used.

**Table 2:** Conventional diagnostics included karyotyping, RT-PCR Panel diagnostics, FISH, and CNV microarray, the latter two as indicated by the treating physician.

Diagnosis (*n*)	Findings through conventional diagnostics (%/cases)	Findings through OGM (%/cases)	Redefinition of karyotype through OGM (%/cases)
AML	61%	87%	70%
AML-MRC	83%	92%	58%
MDS	86%	86%	57%
Total	71%	88%	64%

OGM not only detected relevant structural variants in more cases than conventional diagnostics, but also detected additional variants in 27 cases, leading to clarification and redefinition of the karyotype.

Based on these data, we recently proposed a next-generation work up applying targeted sequencing and a cytogenomics or structural variant profiling approach [5]. This approach does not only identify single nucleotide variants addressable for risk stratification or for targeted therapy such as *NPM1, IDH1*, or *IDH2*. It gives a detailed picture of structural variants by OGM as a single method, allowing to further refine risk stratification and subsequently personalize treatment. Furthermore, this unbiased method with a limited need for time-consuming data interpretation helps discover so far unknown structural variants. In an exemplary case, we were able to identify a *DDX3X::MLLT10* fusion which might play a causative role in the development of this AML. Additionally, the combination of methods uncovered a genetically biphenotypic disease, which was classified as AML with recurrent genetic aberrations by standard diagnostic procedures [5].

We want to thank Yang and colleagues for analyzing this remarkable single-center cohort and adding evidence to our previous work. We think optical genome mapping can be of relevant diagnostic importance not only in MDS and AML patients but also in a variety of hematological malignancies [6, 7].

## Data Availability

The initial OGM output data and further supporting data are available under http://zenodo.org under the accession number 5602040. Further OGM output and raw data supporting this work are available from the corresponding author upon request.
